# A Highly Uncommon Presentation: A Consecutive Three-Case Series of Malignant Ureteral Obstruction in Long-Term Survivors of Non-small Cell Lung Cancer

**DOI:** 10.7759/cureus.100597

**Published:** 2026-01-02

**Authors:** Tomoyuki Araya, Toshiyuki Kita, Takayuki Higashi, Ryo Hara, Hazuki Takato

**Affiliations:** 1 Respiratory Medicine, National Hospital Organization (NHO) Kanazawa Medical Center, Kanazawa, JPN

**Keywords:** advanced non-small cell lung cancer, egfr mutations, long term survivor, malignant ureteral obstruction, ureteral stent

## Abstract

Malignant ureteral obstruction (MUO) is an exceptionally rare complication in non-small cell lung cancer (NSCLC), particularly among long-term survivors. We report three cases of MUO arising in patients with stage IV or recurrent NSCLC, all of whom had survived 3.6-4.0 years after their initial diagnosis. Two of the three cases harbored epidermal growth factor receptor (EGFR) mutations, and MUO in EGFR-mutated NSCLC has not been previously described, suggesting a potential association with prolonged survival under targeted therapy. All patients developed hydronephrosis and renal dysfunction requiring ureteral stent placement. Their clinical courses diverged: one EGFR-mutated patient maintained preserved renal function and remained free from urinary tract infection for three years and four months following stent placement, whereas the remaining two patients experienced rapid disease progression and died within months. These cases indicate that prompt recognition of MUO and early ureteral decompression may meaningfully improve symptoms and preserve renal function, thereby contributing to favorable clinical trajectories in selected long-term survivors. As patient survival continues to lengthen with modern systemic therapies, optimizing drainage strategies will become increasingly important.

## Introduction

Malignant ureteral obstruction (MUO) occurs when tumor invasion or extrinsic compression compromises ureteral patency, resulting in hydronephrosis, renal dysfunction, and, in some cases, urinary tract infection [1,2]. MUO is widely recognized as a manifestation of advanced or terminal-stage malignancy, and previous studies consistently report its profoundly poor prognosis, with median survival generally ranging from approximately three to seven months [1-4]. Because untreated obstruction can lead to irreversible renal injury and may limit the feasibility of systemic therapy, prompt decompression with ureteral stenting or percutaneous nephrostomy is essential for preserving renal function and enabling continuation of cancer treatment [1-3].

Although MUO is frequently encountered in patients with advanced pelvic malignancies or retroperitoneal lymph node metastases, most commonly reported in cancers of gynecologic, gastrointestinal, or urological origin, its occurrence in lung cancer is exceedingly rare, with only a few cases reported in the literature. To better characterize this uncommon manifestation of lung cancer, we report three cases of MUO with the aim of clarifying its clinical features and underlying pathophysiology, thereby providing insights that may support improved patient management.

## Case presentation

Case 1

An 80-year-old woman with advanced lung adenocarcinoma, clinically staged as cT2aN3M1c, stage IVB, harboring an epidermal growth factor receptor (EGFR) exon 19 deletion and a programmed cell death ligand-1 (PD-L1) tumor proportion score (TPS) of 80-90%, diagnosed three years and seven months earlier, presented with fever, right flank pain, and gross hematuria.

Tumor staging was performed according to the 8th edition of the Tumor, Nodes, and Metastasis (TNM) Classification for lung cancer, as outlined by the International Association for the Study of Lung Cancer (IASLC) [5]. This classification system is freely available for academic and clinical use with appropriate citation. The EGFR mutation status was assessed using the cobas® EGFR Mutation Test v2, a validated polymerase chain reaction (PCR)-based companion diagnostic assay [6]. The PD-L1 TPS was determined using the 22C3 anti-PD-L1 antibody according to established immunohistochemical criteria [7]. 

Her medical history included atrial fibrillation at age 76, with no notable family history, smoking history, or allergies. She had received erlotinib, gefitinib, pemetrexed, and continued afatinib beyond progression, maintained on 10 mg daily at presentation. At this point, she had known metastases to the brain, adrenal gland, and intra-abdominal lymph nodes.

On admission, she was febrile (38.1°C) with otherwise stable vital signs and had an Eastern Cooperative Oncology Group performance status (ECOG PS) of 2, as assessed according to the original criteria described by Oken et al. [8]. Physical examination revealed right costovertebral angle tenderness. Laboratory evaluation showed leukocytosis (WBC: 13,400/mm³), elevated C-reactive protein (CRP) (15.31 mg/dL), and impaired renal function (blood urea nitrogen (BUN): 29.7 mg/dL; creatinine: 1.77 mg/dL) (Table 1). Urinalysis demonstrated pyuria and substantial hematuria (Table 1).

**Table 1:** Laboratory findings on admission in case 1 WBC: white blood cell; Neu: neutrophil; Lym: lymphocyte; Mono: monocyte; Eos: eosinophil; Baso: basophil; RBC: red blood cell; Hb: hemoglobin; Hct: hematocrit; Plt: platelet; CRP: C-reactive protein; T-Bil: total bilirubin; TP: total protein; Alb: albumin; ALP: alkaline phosphatase; AST: aspartate aminotransferase; ALT: alanine aminotransferase; γ-GTP: gamma‑glutamyl transpeptidase; LDH: lactate dehydrogenase; Na: sodium; K: potassium; Cl: chloride; BUN: blood urea nitrogen; Cre: creatinine; eGFR: estimated glomerular filtration rate; UA: uric acid; CK: creatine kinase; Amy: amylase; CEA: carcinoembryonic antigen; CYFRA: cytokeratin‑19 fragment; HPF: high‑power field Comprehensive laboratory data obtained at the time of hospital admission in Case 1, demonstrating leukocytosis with neutrophil predominance, marked elevation of inflammatory markers, anemia, hypoalbuminemia, impaired renal function, and urinalysis findings consistent with urinary tract infection. Reference ranges are shown in the rightmost column for comparison

Parameter (Unit)	Value	Reference Range
WBC (/µL)	13400	4500-9000
Neu (%)	88.0	38-74
Lym (%)	3.0	16.5-49.5
Mono (%)	9.0	5-10
Eos (%)	0	0-10
Baso (%)	0	0-2
RBC (×10⁴/µL)	250	382-500
Hb (g/dL)	7.3	11.7-14.6
Hct (%)	21.3	34.3-44.2
Plt (×10⁴/µL)	28.5	15-35
CRP (mg/dL)	15.31	0-0.4
T-Bil (mg/dL)	0.9	0.3-1.2
TP (g/dL)	6.8	6.7-8.3
Alb (g/dL)	2.2	4.0-5.0
ALP (U/L)	566	115-359
AST (U/L)	28	13-33
ALT (U/L)	9	6-27
γ-GTP (IU/L)	94	10-47
LDH (U/L)	149	119-229
Na (mEq/L)	132	135-149
K (mEq/L)	5.5	3.5-4.9
Cl (mEq/L)	97	96-108
BUN (mg/dL)	29.7	8-22
Cre (mg/dL)	1.77	0.5-0.8
eGFR (mL/min)	21.8	60-100
UA (mg/dL)	4.4	2.3-7.0
CK (U/L)	13	45-163
Amy (U/L)	121	35-140
D-dimer (µg/mL)	2.2	0-1
CEA (ng/mL)	2.3	<3.5
CYFRA (ng/mL)	4.4	<3.5
Urinalysis		
pH	7.0	5.0-7.5
Specific gravity	1.008	1.002-1.030
Protein	1+	negative
Glucose	-	negative
Occult blood	3+	negative
Nitrite	-	negative
Urine WBC (qualitative)	3+	negative
Urine WBC (/HPF)	>100	0-4
Urine RBC (/HPF)	>100	0-4

**Figure 1:**
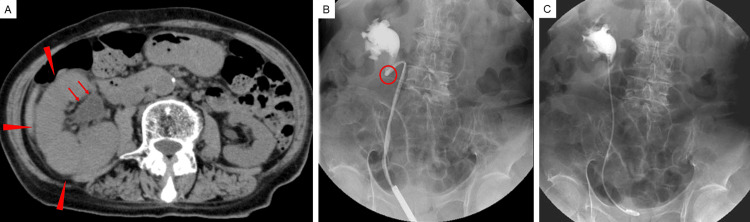
Radiological findings of right-sided hydronephrosis caused by retroperitoneal dissemination and its improvement after double-J stent placement (A) Noncontrast abdominal computed tomography showing right renal enlargement (arrowheads) and hydronephrosis (arrows), accompanied by retroperitoneal dissemination surrounding the right kidney. No direct ureteral invasion was identified. (B) Retrograde pyelography demonstrating no fixed ureteral obstruction; however, kinking at the ureteropelvic junction (circle) caused by retroperitoneal dissemination is evident. (C) The kinking was successfully relieved after smooth, resistance-free placement of a polymer double-J stent (6 Fr, 22 cm)

Urine cytology was negative, while urine and blood cultures both yielded *Escherichia coli*, confirming urosepsis. Antimicrobial therapy was initiated, but her fever persisted. Noncontrast abdominal computed tomography demonstrated right renal enlargement and hydronephrosis accompanied by retroperitoneal dissemination surrounding the right kidney (Figure 1A). Retrograde pyelography revealed kinking at the ureteropelvic junction caused by retroperitoneal tumor involvement (Figure 1B). A 6 Fr, 22 cm polymer double-J stent was placed smoothly and without resistance (Figure 1C), resulting in immediate relief of the obstruction and rapid resolution of the previously antibiotic-refractory fever.

The stent remained functional and was periodically exchanged, allowing preservation of renal function and effective prevention of further infectious complications for the subsequent three years and four months, with only one episode of pyelonephritis occurring nine months after the initial placement. She ultimately died of lung cancer at the end of this period.

Case 2

A 67-year-old woman with lung adenocarcinoma, clinically staged as cT4N3M1c, stage IVB, harboring an EGFR exon 19 deletion and a PD-L1 TPS of <1%, was initially diagnosed five years and eight months earlier and treated surgically. Disease recurrence occurred four years prior to the current presentation. Tumor staging was performed according to the 8th edition of the TNM Classification for lung cancer, as outlined by the IASLC [5]. This classification system is freely available for academic and clinical use with appropriate citation.

The EGFR mutation status was assessed using the cobas® EGFR Mutation Test v2, a validated PCR-based companion diagnostic assay [6]. The PD-L1 TPS was determined using the 22C3 anti-PD-L1 antibody according to established immunohistochemical criteria [7].

She had no significant medical or family history, no allergies, and a smoking history of 20 cigarettes per day for 30 years, having quit 10 years earlier. She had undergone multiple systemic therapies, including osimertinib, carboplatin plus pemetrexed plus pembrolizumab, S-1, docetaxel plus ramucirumab, and nanoparticle albumin-bound paclitaxel, and was taking afatinib 10 mg daily at presentation. At this point, she had known metastases to the lung, pleura, liver, and intra-abdominal lymph nodes.

She presented with acute right lower abdominal pain and nausea. She was afebrile with otherwise stable vital signs, though right costovertebral angle tenderness was present; her ECOG PS was 2, as assessed according to the original criteria described by Oken et al. [8]. Laboratory data showed mild renal dysfunction (BUN 18.9 mg/dL, creatinine 1.18 mg/dL; previously 0.63 mg/dL one week earlier), WBC 4,100/mm³, and CRP 0.69 mg/dL. Urinalysis revealed hematuria without pyuria, and urine culture and cytology were negative (Table 2).

**Table 2:** Laboratory findings on admission in case 2 WBC: white blood cell; Neu: neutrophil; Lym: lymphocyte; Mono: monocyte; Eos: eosinophil; Baso: basophil; RBC: red blood cell; Hb: hemoglobin; Hct: hematocrit; Plt: platelet; CRP: C-reactive protein; T-Bil: total bilirubin; TP: total protein; Alb: albumin; ALP: alkaline phosphatase; AST: aspartate aminotransferase; ALT: alanine aminotransferase; γ-GTP: gamma‑glutamyl transpeptidase; LDH: lactate dehydrogenase; Na: sodium; K: potassium; Cl: chloride; BUN: blood urea nitrogen; Cre: creatinine; eGFR: estimated glomerular filtration rate; UA: uric acid; CK: creatine kinase; Amy: amylase; CEA: carcinoembryonic antigen; CYFRA: cytokeratin‑19 fragment; HPF: high‑power field Comprehensive laboratory findings obtained at the time of admission in Case 2 demonstrated mild leukopenia, normal inflammatory markers except for slightly elevated CRP, mild anemia, hypoalbuminemia, impaired renal function, and tumor marker elevation. Urinalysis showed acidic urine with proteinuria (±) and marked hematuria without pyuria. Reference ranges are provided for comparison

Parameter (unit)	Value	Reference range
WBC (/µL)	4100	4500-9000
Neu (%)	75.3	38-74
Lym (%)	12.6	16.5-49.5
Mono (%)	7.4	5-10
Eos (%)	4.2	0-10
Baso (%)	0.5	0-2
RBC (×10⁴/µL)	346	382-500
Hb (g/dL)	10.4	11.7-14.6
Hct (%)	33.6	34.3-44.2
Plt (×10⁴/µL)	24.5	15-35
CRP (mg/dL)	0.69	0-0.4
T-Bil (mg/dL)	0.5	0.3-1.2
TP (g/dL)	5.8	6.7-8.3
Alb (g/dL)	3.2	4.0-5.0
ALP (U/L)	307	115-359
AST (U/L)	16	13-33
ALT (U/L)	11	6-27
γ-GTP (IU/L)	20	10-47
LDH (U/L)	200	119-229
Na (mEq/L)	142	135-149
K (mEq/L)	4.6	3.5-4.9
Cl (mEq/L)	104	96-108
BUN (mg/dL)	18.9	8-22
Cre (mg/dL)	1.18	0.5-0.8
eGFR (mL/min)	35.8	60-100
UA (mg/dL)	2.8	2.3-7.0
CK (U/L)	76	45-163
Amy (U/L)	68	35-140
D-dimer (µg/mL)	1.0	0-1
CEA (ng/mL)	59.2	<3.5
CYFRA (ng/mL)	3.7	<3.5
Urinalysis		
pH	6.5	5.0-7.5
Specific gravity	1.029	1.002-1.030
Protein	±	negative
Glucose	-	negative
Occult blood	3+	negative
Nitrite	-	negative
Urine WBC (qualitative)	3+	negative
Urine WBC (/HPF)	1-4	0-4
Urine RBC (/HPF)	>100	0-4

**Figure 2:**
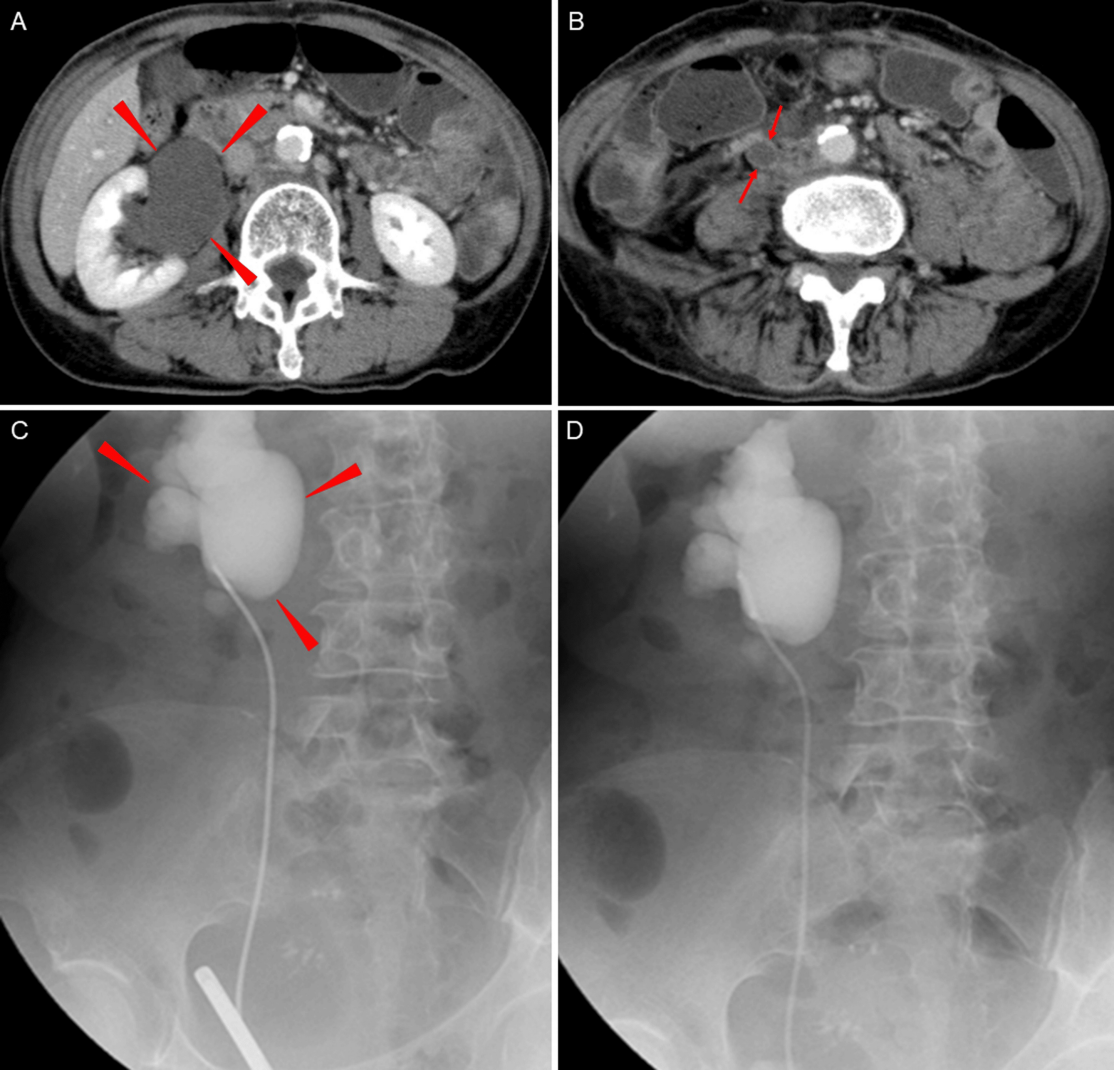
Radiological findings of advanced hydronephrosis and ureteral involvement caused by retroperitoneal dissemination, and its management with double-J stent placement (A) Contrast-enhanced abdominal computed tomography showing marked right hydronephrosis and hydroureter (arrowheads), accompanied by retroperitoneal dissemination surrounding the kidney. (B) The ureter coursing through the area of retroperitoneal dissemination demonstrates long-segment ureteral wall thickening and luminal narrowing (arrows). (C) Retrograde pyelography revealing no fixed obstruction of the right ureter but demonstrating marked hydronephrosis (arrows). Based on these findings, malignant ureteral obstruction due to retroperitoneal dissemination was diagnosed. (D) A 24 cm, 6 Fr double-J stent was placed smoothly and without resistance, resulting in appropriate drainage

Contrast-enhanced abdominal CT demonstrated marked right hydronephrosis and hydroureter accompanied by retroperitoneal dissemination surrounding the right kidney, in addition to previously noted abdominal lymph node metastases (Figure 2A). Along the portion of the ureter coursing through the area of retroperitoneal dissemination, contrast-enhanced CT showed long-segment ureteral wall thickening and luminal narrowing (Figure 2B). Retrograde pyelography revealed no fixed obstruction of the right ureter but demonstrated marked hydronephrosis (Figure 2C). Based on these findings, MUO secondary to retroperitoneal dissemination was diagnosed. A 6-Fr, 24-cm polymer double-J stent was placed smoothly and without resistance (Figure 2D), resulting in rapid improvement in pain, hydronephrosis, and renal function.

Adverse events were limited to bladder irritation and one urinary tract infection. Despite successful decompression, her cancer progressed rapidly, and she died two months later.

Case 3

A 55-year-old man with stage IVB lung adenocarcinoma, clinically staged as cT2bN3M1c, with a PD-L1 TPS of 80-90% and no actionable driver mutations identified on prior testing, diagnosed three years and seven months earlier, presented with left flank pain and fatigue. Tumor staging was performed according to the 8th edition of the TNM Classification for lung cancer, as outlined by the IASLC [5]. This classification system is freely available for academic and clinical use with appropriate citation.

The EGFR mutation status and other actionable genomic alterations were assessed using the Oncomine Dx Target Test, a next-generation sequencing-based companion diagnostic assay validated in real-world clinical practice [9]. The PD-L1 TPS was determined using the 22C3 anti-PD-L1 antibody according to established immunohistochemical criteria [7].

His medical history included neurofibromatosis type I at age 52, with no family or allergy history, and a smoking history of 20 cigarettes per day for 40 years. He had received gamma-knife radiosurgery and additional stereotactic irradiation for brain metastases, followed by systemic therapies including carboplatin plus pemetrexed plus pembrolizumab, docetaxel plus ramucirumab, and S-1, before transitioning to best supportive care after disease progression. At this point, he had known metastases to the lung, pleura, brain, bone, liver, adrenal glands, and retroperitoneum.

He was afebrile with otherwise stable vital signs, though left costovertebral angle tenderness was noted; his ECOG PS was 3, as assessed according to the original criteria described by Oken et al. [8]. Laboratory tests demonstrated elevated inflammatory markers (WBC: 9,900/mm³; CRP: 4.67 mg/dL) and worsening renal function (BUN: 31.3 mg/dL; creatinine: 2.40 mg/dL, increased from 1.73 mg/dL one week earlier). Urinalysis was unremarkable, and urine culture and cytology were negative (Table 3).

**Table 3:** Laboratory findings on admission in case 3 WBC, white blood cell; Neu, neutrophil; Lym, lymphocyte; Mono, monocyte; Eos, eosinophil; Baso, basophil; RBC, red blood cell; Hb, hemoglobin; Hct, hematocrit; Plt, platelet; CRP, C-reactive protein; T-Bil, total bilirubin; TP, total protein; Alb, albumin; ALP, alkaline phosphatase; AST, aspartate aminotransferase; ALT, alanine aminotransferase; γ-GTP, gamma-glutamyl transpeptidase; LDH, lactate dehydrogenase; Na, sodium; K, potassium; Cl, chloride; BUN, blood urea nitrogen; Cre, creatinine; eGFR, estimated glomerular filtration rate; UA, uric acid; CK, creatine kinase; Amy, amylase; CEA, carcinoembryonic antigen; CYFRA, cytokeratin-19 fragment; HPF, high-power field Laboratory data obtained at admission in case 3 showed normocytic anemia, mild leukocytosis, impaired renal function, and modest inflammatory activity. Liver function tests were largely within the normal range except for elevated γ-GTP. Urinalysis demonstrated normal pH, normal specific gravity, and no evidence of proteinuria, glycosuria, hematuria, or pyuria. All parameters and reference ranges are summarized in the table

Parameter (unit)	Value	Reference range
WBC (/µL)	9900	4500-9000
Neu (%)	70.1	38-74
Lym (%)	11.6	16.5-49.5
Mono (%)	9.6	5-10
Eos (%)	7.3	0-10
Baso (%)	1.4	0-2
RBC (×10⁴/µL)	315	414-575
Hb (g/dL)	7.7	13.0-17.1
Hct (%)	26.0	39.6-50.8
Plt (×10⁴/µL)	38.8	15-35
CRP (mg/dL)	4.67	0-0.4
T-Bil (mg/dL)	0.4	0.3-1.2
TP (g/dL)	6.0	6.7-8.3
Alb (g/dL)	3.0	4.0-5.0
ALP (U/L)	540	115-359
AST (U/L)	22	13-33
ALT (U/L)	25	8-42
γ-GTP (IU/L)	166	10-47
LDH (U/L)	172	119-229
Na (mEq/L)	135	135-149
K (mEq/L)	5.2	3.5-4.9
Cl (mEq/L)	103	96-108
BUN (mg/dL)	31.3	8-22
Cre (mg/dL)	2.40	0.6-1.0
eGFR (mL/min)	23.6	60-100
UA (mg/dL)	6.0	3.6-7.0
CK (U/L)	27	45-163
Amy (U/L)	58	35-140
D-dimer (µg/mL)	2.1	0-1
CEA (ng/mL)	2.2	<3.5
CYFRA (ng/mL)	7.0	<3.5
Urinalysis		
pH	7.5	5.0-7.5
Specific gravity	1.012	1.002-1.030
Protein	-	negative
Glucose	-	negative
Occult blood	-	negative
Nitrite	-	negative
Urine WBC (qual)	-	negative
Urine WBC (/HPF)	<1	0-4
Urine RBC (/HPF)	<1	0-4

**Figure 3:**
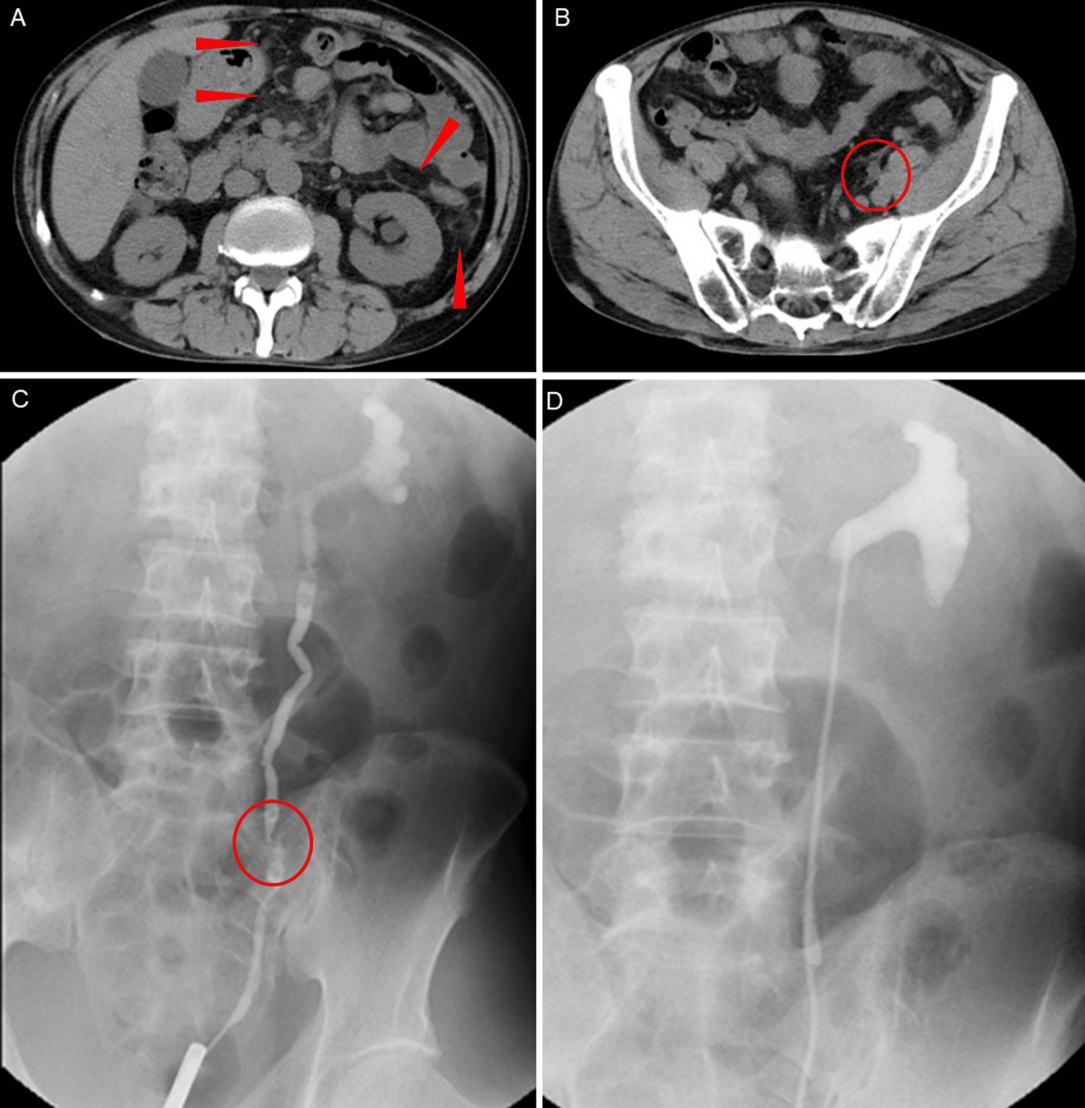
Radiological findings of left-sided malignant ureteral obstruction caused by retroperitoneal dissemination and its improvement after metallic ureteral stent placement (A) Noncontrast abdominal computed tomography showing left hydronephrosis and retroperitoneal fat stranding due to retroperitoneal dissemination (arrowheads). (B) In the pelvis, a soft-tissue density lesion is seen along the left ureter (circle), raising suspicion of impaired urinary flow caused by retroperitoneal dissemination. (C) Retrograde pyelography demonstrating a focal stricture of the mid-left ureter (circle), consistent with malignant ureteral obstruction secondary to retroperitoneal dissemination. (D) After administering a sacral block, a metallic ureteral stent (tumor stent, 7 Fr, 22 cm) was placed using a rigid cystoscope. A sensor guidewire initially failed to pass through the stricture; however, an angled hydrophilic guidewire was successfully advanced across the lesion, which subsequently allowed passage of the sensor guidewire

Two months after initiating supportive care, noncontrast abdominal CT demonstrated worsening retroperitoneal dissemination with newly developed left hydronephrosis (Figures 3A-3B). Retrograde pyelography revealed a focal mid-ureteral stricture consistent with MUO secondary to retroperitoneal dissemination (Figure 3C). Following a sacral block, a 7 Fr, 22 cm metallic ureteral stent was placed using a rigid cystoscope. A sensor guidewire initially failed to cross the stricture, but an angled hydrophilic guidewire successfully traversed the lesion, allowing subsequent passage of the sensor guidewire and completion of stent placement (Figure 3D).

Following stent placement, hydronephrosis, renal function, and flank pain improved. He died three months later due to the progression of lung cancer.

## Discussion

Our three cases highlight a striking and clinically important observation: MUO can arise even in long-term survivors of advanced lung cancer. All patients developed MUO despite having stage IV disease or postoperative recurrence and surviving as long-term survivors, with survival durations ranging from 3.6 to 4.0 years after their initial cancer diagnosis. Interestingly, two of the three cases harbored EGFR mutations, and to our knowledge, MUO in EGFR-mutated lung cancer has not been previously reported, suggesting that this complication may represent a rare manifestation in a population that typically achieves prolonged survival with targeted therapies. This finding further underscores a unique biological characteristic of EGFR-mutated tumors, namely, their capacity for long-term disease control while retaining the potential to metastasize to uncommon sites [10]. These observations indicate that MUO, although exceedingly rare in lung cancer, may occur late in the disease course among patients who benefit from durable systemic therapy, a clinical scenario not widely recognized.

Previous studies have reported that MUO typically develops in the terminal stages of malignancy, with a median survival ranging from approximately three to seven months after its onset [1-4]. However, one of our patients survived for three years and four months following ureteral stent placement, suggesting that preservation of renal function and effective control of urinary tract infection may have contributed positively to her overall clinical trajectory. This case highlights the potential value of early stent placement in selected patients, even when MUO develops during advanced disease. In the remaining two cases, extended survival was not achieved; nevertheless, both individuals experienced meaningful symptomatic improvement, including relief from flank pain and fever, demonstrating the important palliative role of ureteral decompression.

The management of MUO generally relies on urinary drainage procedures, most commonly polymeric ureteral stents. Although widely used, polymeric stents are vulnerable to external tumor compression and often require replacement every three months due to occlusion. Metallic ureteral stents, which offer greater resistance to extrinsic pressure, provide an alternative with potentially longer patency and may contribute to better preservation of renal function and maintenance of quality of life in selected patients [11,12]. However, metallic ureteral stents have notable drawbacks, including increased discomfort during insertion or exchange and the frequent need for anesthesia [13]. They are also substantially more expensive than polymeric stents, and cost considerations may influence treatment decisions, particularly in patients with limited life expectancy. Consequently, current clinical practice involves selecting polymeric or metallic ureteral stents on an individualized basis, taking into account the patient’s general condition, anticipated prognosis, technical feasibility, cost, and personal preference, with the goal of balancing durability, procedural burden, and quality of life [14]. In our three cases, all patients were unexpectedly long-term survivors, making prognosis estimation at the time of MUO diagnosis inherently difficult. Therefore, for two patients, polymeric ureteral stents were selected because they offered adequate drainage with minimal procedural burden. In contrast, in one patient with end-stage disease and extensive retroperitoneal involvement, a metallic mesh tumor ureteral stent was chosen to maximize luminal patency; however, despite its durability, this option carried disadvantages, including the need for sacral nerve block during placement and a substantially higher cost. In all cases, the type of ureteral stent was determined through thorough shared decision-making between the urologists and the patient, ensuring that individual preferences and clinical priorities were appropriately balanced.

Taken together, our findings emphasize that respiratory physicians should remain vigilant for MUO as a rare but important complication in long-term survivors of lung cancer. When renal dysfunction is observed, prompt evaluation for MUO and early consideration of ureteral stent placement may help preserve renal function, improve symptoms, and, in selected cases, contribute to favorable clinical outcomes. Awareness of this uncommon entity is becoming increasingly important in the era of prolonged survival among patients with advanced lung cancer, particularly those with EGFR-mutated disease.

This case series represents consecutive cases from a single institution; however, the timing and presentation of symptoms, as well as clinical decision-making, differed among patients. Therefore, similar cases may have remained undetected. In addition, the small number of cases and the heterogeneity of patient backgrounds limit generalizability. No statistical analysis was performed, as this report was intended as a descriptive case series.

## Conclusions

MUO is an exceptionally rare complication in lung cancer, yet our three cases demonstrate that it can arise even in long-term survivors, including those with EGFR-mutated disease in whom MUO has not been previously reported. These findings highlight that MUO may develop late in the disease course among patients receiving prolonged systemic therapy and underscore the importance of prompt recognition when renal dysfunction occurs. Early ureteral stent placement may help preserve renal function, relieve symptoms, and, in selected patients, contribute to improved clinical outcomes. As survival continues to improve for patients with advanced lung cancer, awareness of this uncommon but clinically significant complication will become increasingly essential for respiratory physicians.
